# Local Signal Time-Series during Rest Used for Areal Boundary Mapping in Individual Human Brains

**DOI:** 10.1371/journal.pone.0036496

**Published:** 2012-05-04

**Authors:** Satoshi Hirose, Takamitsu Watanabe, Koji Jimura, Masaki Katsura, Akira Kunimatsu, Osamu Abe, Kuni Ohtomo, Yasushi Miyashita, Seiki Konishi

**Affiliations:** 1 Department of Physiology, The University of Tokyo School of Medicine, Bunkyo-ku, Tokyo, Japan; 2 Department of Radiology, The University of Tokyo School of Medicine, Bunkyo-ku, Tokyo, Japan; Hangzhou Normal University, China

## Abstract

It is widely thought that resting state functional connectivity likely reflects functional interaction among brain areas and that different functional areas interact with different sets of brain areas. A method for mapping areal boundaries has been formulated based on the large-scale spatial characteristics of regional interaction revealed by resting state functional connectivity. In the present study, we present a novel analysis for areal boundary mapping that requires only the signal timecourses within a region of interest, without reference to the information from outside the region. The areal boundaries were generated by the novel analysis and were compared with those generated by the previously-established standard analysis. The boundaries were robust and reproducible across the two analyses, in two regions of interest tested. These results suggest that the information for areal boundaries is readily available inside the region of interest.

## Introduction

Resting state functional connectivity, measures of correlation of low-frequency BOLD signal between brain regions during resting [1]–[4], has revealed strong functional correlation between distant brain regions [5]–[13]. Unlike diffusion tensor imaging (DTI) tractography which measures the directional diffusion of water within a voxel and reveals direct anatomical connections [14]–[17], the linkage between highly correlated regions can evidently be indirect [18]. Although the neural mechanisms for the low-frequency fluctuations in the BOLD signal are not fully understood, the robust functional correlation between regions that tend to be co-activated during particular cognitive processing is thought to reflect interaction between brain regions, thus constituting large-scale regional networks [19]–[27].

Recent evidence has shown that resting state functional connectivity can be used to delineate boundaries of functionally distinct areas [28]. Information as to which part of the brain is functionally correlated with a particular location in a region of interest can be obtained by calculating voxel-wise functional connectivity when a seed is placed at a location in that region. Movement of the seed from the original location sometimes encounters an abrupt change in a spatial pattern of voxel-wise correlation in the whole brain, which is thought to reflect boundaries between areas for distinct functions implemented by distinct large-scale regional networks. A standard boundary mapping method was formulated based on this observation [18], [29], and successful applications of the method have been reported in several areas such as the cingulate areas [18], [28], the supramarginal/angular gyrus [18], the lateral parietal cortex [29] and various regions in the whole brain [30], [31].

The standard boundary mapping method defines areal boundaries between adjacent areas by referring to information from the whole brain. In the present study, we present a new analysis for boundary mapping in which areal boundaries are delineated based on the BOLD signal timecourse within these adjacent areas, without information taken from the other part of the brain. More specifically, we reasoned that signal timecourse that is supposed to be relatively uniform within an area should be changed abruptly across the boundary between adjacent areas. The approach of utilizing the local timecourse information would exempt the relatively complicated procedures required in the standard method, including calculation of whole-brain functional correlation and detection of the abrupt change in a spatial pattern of the whole-brain functional correlation. We show areal boundaries drawn by the new analysis, and examine the validity of the new method by comparing the results obtained from these two methods. These two methods may yield different patterns of areal boundaries, but we report remarkable similarity between them.

## Methods

### Data Acquisition

Structural and functional images were collected at a 3 T MRI system. Functional images during the resting state were collected using gradient echo echo-planar sequences (TR = 6.0 sec, TE = 35 msec, flip angle = 90 deg, cubic voxel of 2 mm, 34 slices without slice gap). The data were sampled using cubic voxels of 2 mm to minimize signal contamination from the other bank of a sulcus [32]–[36]. Each run contained 54 volume images, and 64 runs were collected for each of the subjects in multiple sessions to compensate for lower signal to noise ratio caused by the small voxel size. The first four images in each run were excluded from the analysis in order to take into account the equilibrium of longitudinal magnetization. Written informed consent was obtained from 3 healthy right-handed subjects (2 males; 1 female, age: 22–28 years). They were scanned by fMRI using experimental procedures approved by the institutional review board of the University of Tokyo School of Medicine. For the population average analysis, functional images of the resting state were provided from the data set of 25 subjects (13 males; 12 females, age: 20–28 years) used in Kimura et al. (2010) [37] (TR = 3.0 sec, TE = 35 msec, flip angle = 90 deg, cubic voxel of 4 mm^3^, 40 slices without slice gap, 2 runs of 100 volumes each). For anatomic reference, T2-weighted images were obtained for spatial normalization (TR = 3 s, TE = 85 ms, 80 slices, slice thickness = 2.0 mm, in-plane resolution = 1×0.67 mm^2^).

### Preprocessing

Functional images were realigned and were slice timing corrected using SPM2 [38]. When head movement occurred by more than 2 mm in translation or by more than 2 degrees in any rotation, the entire run was excluded (one run from one subject). Although slice timing correction may be less accurate in a longer TR, we followed the standard method [18], [21] and had to conduct slice timing correction because the timecourses had to be averaged across voxels in the white matter, ventricle and whole brain to regress out nuisance signals. However, the comparison between the standard and new boundary mapping methods in the present study will not be influenced by the slice timing correction since the procedures were used commonly in the two methods. Because the functional images were acquired in the spatial resolution of 2×2×2 mm, spatial smoothing was not conducted to keep the functional images from being blurred. Spatial normalization was not applied either to avoid spatial smoothing included in the normalization algorithm, and the whole analyses were conducted in individual subjects. The images realigned and slice timing corrected were subject to temporal band-pass filtering (0.009 Hz<f<0.08 Hz) using FSL [39]. The filtered images were further subject to regression using SPM2 based on a general linear model [38], [40] with parameters obtained by head motion correction, whole brain signal averaged over the whole brain, ventricular signal averaged from ventricular ROI, and white matter signal averaged from white matter ROI [21], [41]. For the population average analysis for 4-mm resolution data, spatial normalization and smoothing (FWHM = 8 mm) were applied.

### Surface-Based Mapping

The posterior part of the inferior frontal cortex (pIFC) in the right hemisphere, a part of the association cortex that implements several well-investigated functions [42]–[45], was a region of central interest in the present study. The right pIFC in each individual subject was analyzed in detail using two-dimensional surface mapping based on Caret (http://brainmap.wustl.edu/caret) [46]. The SureFit method was applied to a functional image of the 2-mm resolution from each individual subject, which resulted in a segmentation whose boundary runs approximately midway through the cortical thickness. The segmentation was used to automatically generate a wire-frame tessellation whose nodes lied on the boundary of the segmentation. The wire-frame tessellation was further inflated, and was flattened by making cuts along five standardized trajectories to allow for inspection of the pIFC in a two dimensional space.

### Generation of Probabilistic Boundary Maps (New Analysis)

**Figure 1:**
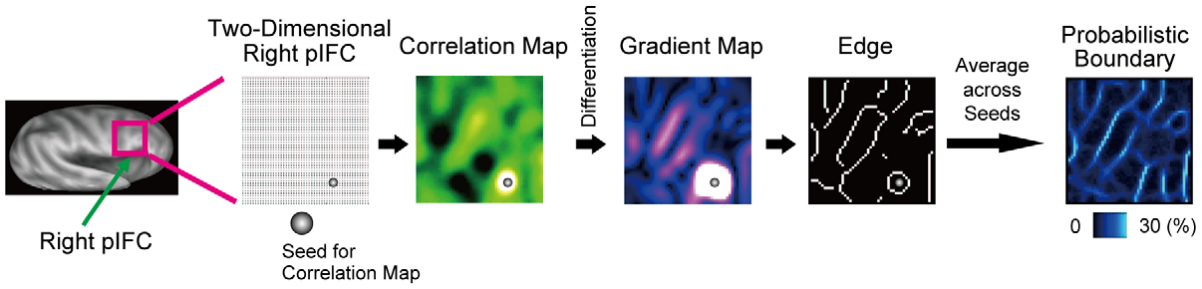
A flow chart of the new boundary mapping method. The region of interest, the right pIFC, was flattened into a two-dimensional space, and was subject to the new boundary mapping analysis.

Rather than the spatial pattern of whole-brain correlation maps, we utilized the local signal timecourses within the region of interest for boundary mapping. Each pixel in the 2D space (50 mm×50 mm) that covered the right pIFC was used as the seed to calculate correlation coefficient of signal timecourses between the seed and all the pixels in the region (Fig. 1). A correlation map in the right pIFC was generated by applying the Fisher's z-transformation to the correlation coefficient [21], [41]. Therefore, the correlation maps were generated by the number of all the seed pixels in the 2D space. The correlation map was analyzed to detect boundaries where the correlation changed most abruptly. Canny edge detection algorithm [47], which also includes spatial differentiation, was applied to the correlation map to generate a gradient map and then to detect edges [18], [29] (Fig. 1). The Canny method smoothes the correlation map with a Gaussian filter (FWHM = 6 mm) to reduce noise, and then creates a gradient map by spatial differentiation of the correlation map. High gradient values in the gradient map represent locations where similarity between the timecourses is abruptly changing. After eliminating pixels in the gradient map that are not local maxima, the algorithm tracks along highlighted regions in the gradient map, which generates an edge map. Since the edge detection is binary, averaging across the entire set of binary edge maps for all the seed pixels generates a probabilistic boundary map in which intensity represents how likely a location is to be an edge (Fig. 1). As an additional reference, regions around the right central sulcus were also subject to the boundary mapping analyses.

### Comparison with Standard Analysis

We also used the standard analysis for boundary mapping [18], [29], which detects pixels where the spatial pattern of whole-brain correlation maps changes abruptly, and compared the results of our new analysis with those of the standard analysis. The procedures for generation of the gradient maps, the edge maps, and the probabilistic boundary maps were common to the standard and the new analysis methods, and the same parameters for edge detection were applied. Moreover, although the standard analysis calculated voxel-wise correlation in the whole brain for each seed, we also restricted the target of correlation calculation: (1) the gray matter in the entire cerebral cortex and (2) the region contralateral to the right pIFC. The use of only a part of the brain as a target of correlation calculation in the standard analysis is expected to decrease the number of slices and calculation cost. The gray matter was segmented in individual subjects using SPM2. The contralateral region was determined as across-seed collection of the voxels in the spherical region (radius: 2 mm) in the contralateral part of right pIFC. The idea is that a region has the strongest functional connectivity with the corresponding contralateral region [48], [49] that may be sufficient for boundary mapping.

To make a quantitative comparison of the signal to noise ratio between the maps by the standard and new methods, we defined a signal to noise ratio. Generally, the signal to noise ratio is defined as the magnitude of signal divided by the magnitude of noise. More specifically, in the present situation, the signal to noise ratio was defined as the average of the probability values in the Boundary pixels, divided by the average of the probability values in the Background pixels. The Boundary and Background pixels were defined as follows: The Boundary pixels were on the ridge of the probabilistic boundary, whereas Background pixels were off that ridge. To define the Boundary pixels, the direction of any one pixel was first determined out of four possible directions by summing up the pixel values of three linear pixels (the pixel itself and two adjacent pixels) along each of the four possible directions and selecting the greatest one. The pixel was regarded as a Boundary pixel (1) when the pixel value was the greatest of the three orthogonal pixels (the pixel itself and the two adjacent pixels lined orthogonally to the selected direction), and (2) when such pixels were contiguous by three or more. The pixel was regarded as a Background pixel when there were no Boundary pixels in any of the eight pixels surrounding that pixel.

## Results

**Figure 2:**
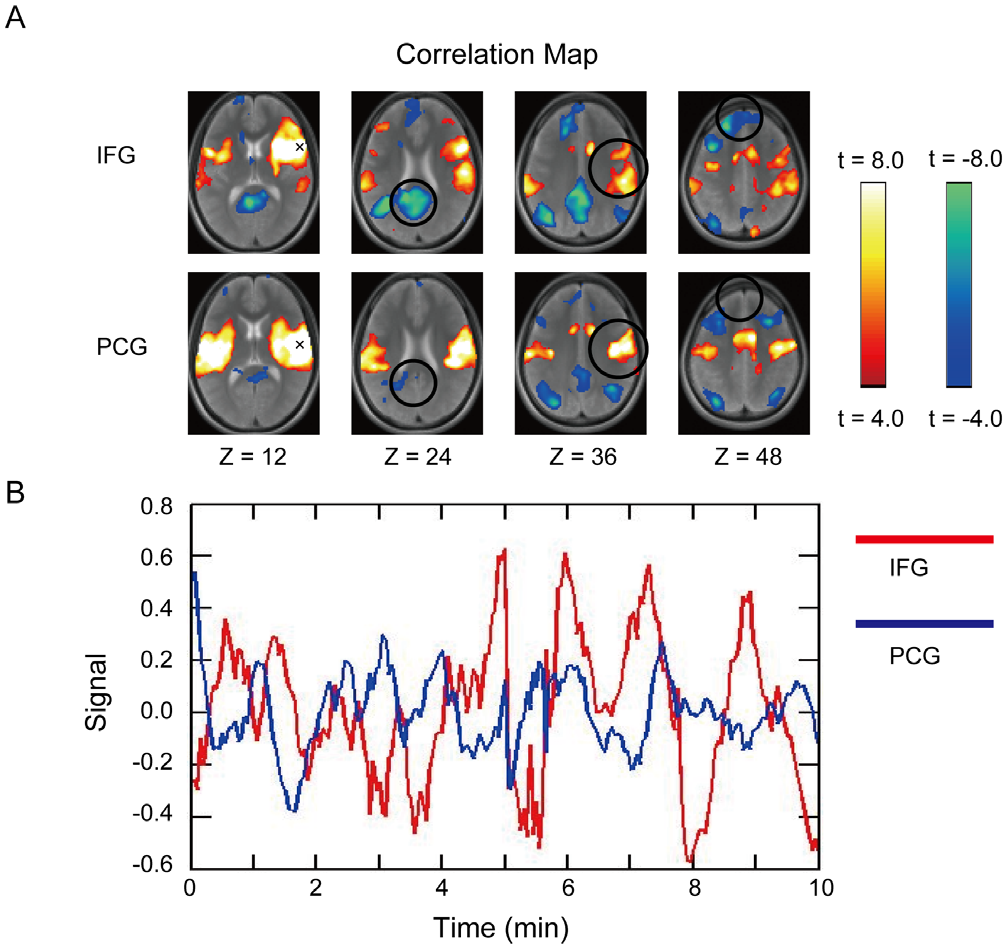
Different spatial patterns in whole-brain correaltion maps and the signal timecourses between the 2 seeds as close as 2 cm. A) Whole-brain correlation maps that exhibited distinct spatial patterns when seeds were placed around the right pIFC about 2 cm apart, at (54, 12, 12) and (54, −8, 12) [51]. A seed is indicated by “x”. IFG: inferior frontal gyrus, PCG: precentral gyrus. B) fMRI signals in the two seed points in one representative subject that exhibited dissimilar timecourses. The correlation between the timecourses was utilized in the present study for boundary mapping.

We first demonstrate that the many parts of the spatial patterns in whole-brain correlation maps can be different even when the two seeds are as close as 2 cm [18]. Figure 2A shows two correlation maps when the seeds were placed in the right pIFC. The signal timecourses in the two seeds were also different, which was utilized for boundary mapping in the present new analysis (Fig. 2B).

**Figure 3:**
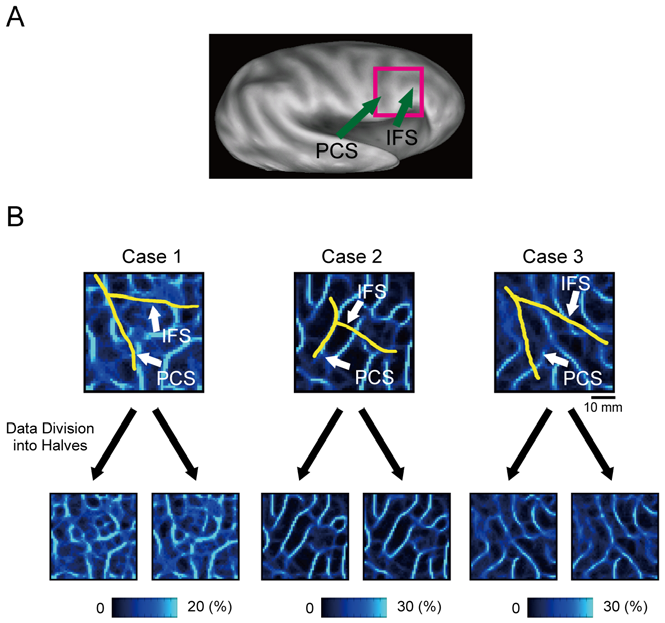
The probabilistic boundary maps around the right pIFC generated by the standard analysis with whole-brain calculation of correlation. A) An inflated brain and the region of interest, the right pIFC. PCS: precentral sulcus, IFS: inferior frontal sulcus. B) Reproducibility of boundary patterns based on the standard mapping method in three individual subjects when data set was divided into two halves (left panels: odd runs, right panels: even runs). Yellow curves indicate the approximate location of the fundus of the PCS or IFS.

The standard boundary mapping method was applied to the right pIFC (Fig. 3A), and the probabilistic boundary maps were generated (Fig. 3B). The sulcus borders (PCS and IFS) were manually drawn in yellow to provide approximate orientation as a reference to the inferior frontal cortex. We tested the reproducibility of the probabilistic boundary maps. When the data from the total of 64 runs were divided into two (32 runs each) by alternately classifying the runs for each subject, the probabilistic boundary maps generated from each of the two data sets exhibited robust boundary patterns. To evaluate the similarity between the boundary maps based on the divided data sets, the correlation coefficients were calculated between the boundary maps in the three cases, with the degrees of freedom corrected by Bartlett correction factor [50]. All of these were highly significant (the lowest r = 0.67, t_(130)_ = 10.2, p = 3.4×10^−18^), demonstrating the reliability of the probabilistic boundary maps generated by the standard analysis.

**Figure 4:**
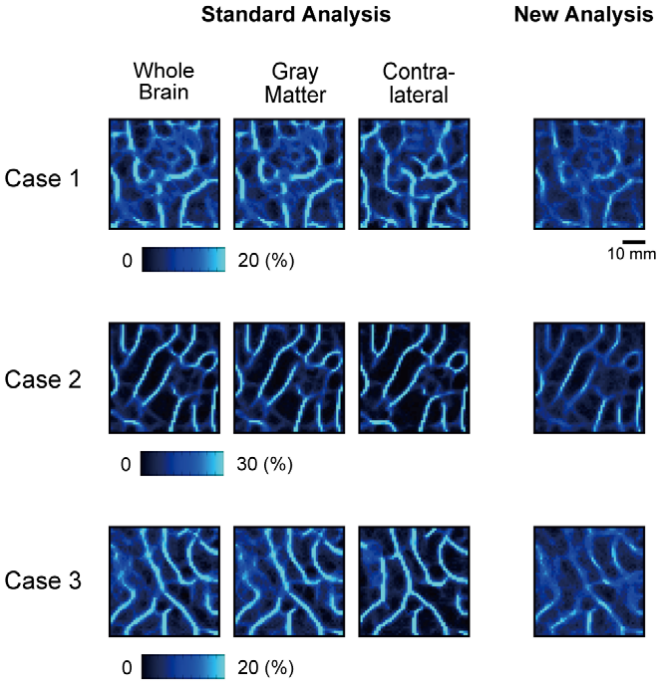
Boundary patterns in the right pIFC generated by the standard analysis (left) and the new analysis (right) in three individual subjects. In the standard analysis, the correlation map was calculated for the whole brain, the gray matter in the entire cerebral cortex, or the region contralateral to the right pIFC.

The range of correlation calculation in the standard boundary mapping method was restricted to only a part of the brain: (1) the gray matter in the entire cerebral cortex and (2) the region contralateral to the right pIFC, rather than the whole-brain. As shown in Fig. 4 left, the restriction of the correlation calculation yielded largely equivalent patterns of probabilistic boundaries. To evaluate the similarity, the correlation coefficients were calculated between the boundary maps based on whole-brain correlation and those based on only a part of the brain in the three cases, and all of these were highly significant (the lowest r = 0.68, t_(124)_ = 10.1, p = 8.0×10^−18^).

The probabilistic boundary maps were also generated using the new boundary mapping method using the same parameters for generation of gradient and edge maps. The new analysis successfully yielded probabilistic boundary patterns, and the boundary patterns were very similar to those generated by the standard analysis (Fig. 4 right). To evaluate the similarity, the correlation coefficients were calculated between the boundary maps based on the standard analysis using the whole-brain correlation maps and those based on the new analysis in the three cases, and all of these were highly significant (the lowest r = 0.88, t_(134)_ = 21.1, p = 3.0×10^−44^). These results confirm the validity of the new analysis method.

To quantify the comparison of the boundary maps yielded by the standard and new methods, we calculated the signal to noise ratio (see Methods) and presented the results in Table 1. The ratio for the boundary map generated by the new method was lowered by approximately 50% relative to that by the standard method with whole-brain calculation of correlation. We also measured computation time taken to generate the boundary maps using a personal computer (Dell Precision T5400, CentOS 5.5, CPU: 2.66 GHz with quad core 2×6 MB L2 cache and 1333 MHz FSB, memory: 32 GB) and Matlab R2007b. Step 1 consisted of generation of correlation maps in the new method and generation of both correlation and eta^2^ maps in the standard method, whereas Step 2 consisted of common procedures of generation of gradient maps, edges and probabilistic boundary maps. As shown in Table 2, the computation time was reduced drastically to approximately one hundredth using the new method, as compared to the standard method with whole-brain calculation of correlation, at the expense of the signal to noise ratio. The computation time in the standard method with correlation calculation only in the contralateral region was also reduced drastically, as compared to the standard method with whole-brain calculation of correlation, without loss of the signal to noise ratio. Despite the lower signal to noise ratio in the new method, however, the results demonstrate that local signals contain information that can be used for boundary mapping.

**Table 1:** Signal-to-noise ratio of probabilistic boundary maps in three cases generated by the standard and new boundary mapping methods.

	Standard Analysis			New Analysis
	Whole Brain	Gray Matter	Contralateral	
Case 1	2.8	2.9	3.7	1.8
Case 2	7.6	6.6	11.2	3.1
Case 3	3.1	3.0	7.6	2.0
Average	4.5	4.2	7.5	2.3

**Table 2:** Calculation time (in minutes) taken to generate probabilistic boundary maps in three cases by the standard and new boundary mapping methods.

	Standard Analysis						New Analysis	
	Whole Brain		Gray Matter		Contralateral			
	Step 1	Step 2	Step 1	Step 2	Step 1	Step 2	Step 1	Step 2
Case 1	2144.8	1.7	813.8	1.8	23.7	1.7	15.0	1.7
Case 2	2085.2	1.7	872.0	1.6	26.5	1.8	14.4	1.7
Case 3	2046.8	1.7	838.0	1.7	22.0	1.8	14.5	1.6
Average	2092.2	1.7	841.3	1.7	24.1	1.7	14.6	1.7

**Figure 5:**
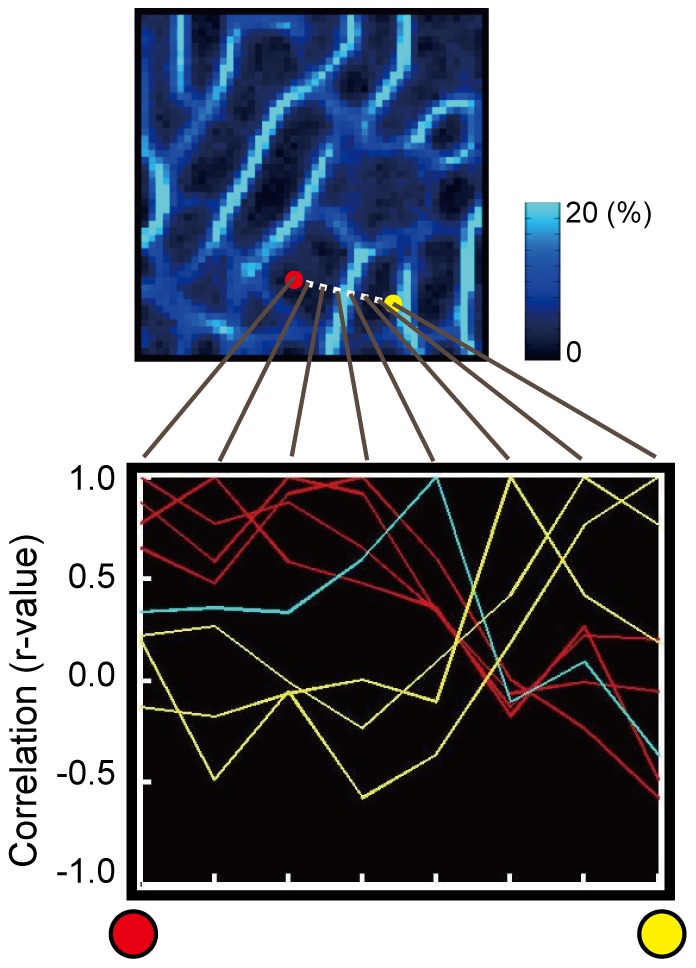
A spatial profile of correlation coefficient between signal timecourses along the line that crossed a representative boundary. The red and yellow curves indicate the spatial profiles that belonged to the same areas as the red and yellow dots, respectively.

To inspect that the pixels that had similar signal timecourses were spatially grouped by the boundaries detected by the new analysis, two pixels were selected across a representative boundary, and the correlation of signal timecourse was examined along the line between the two pixels, which included 8 pixels in total. The correlation of signal timecourses between one pixel and each of all the 8 seed pixels along this line were calculated, yielding a spatial profile that peaked at the given seed pixel. As shown in Fig. 5, the boundary delineated the region into two parts, each of which consisted of pixels of similar profiles of correlation coefficient, demonstrating that the signal timecourse was similar within each of the delineated areas, but not across the probabilistic boundary.

**Figure 6:**
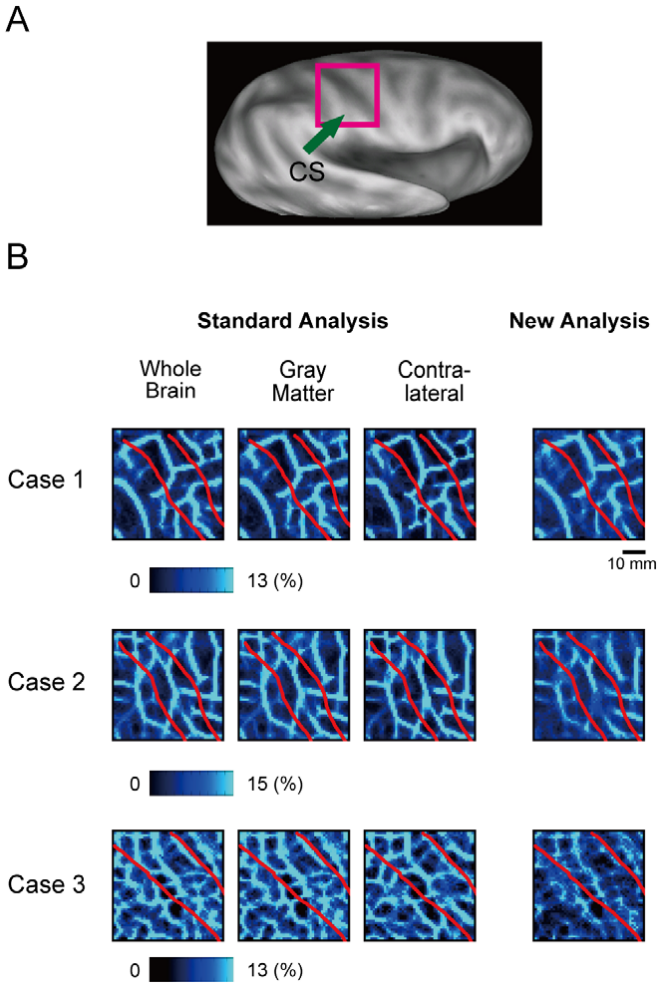
The probabilistic boundary maps around the right central sulcus. A) An inflated brain and the region of interest, the right central sulcus. CS: central sulcus. B) Boundary patterns in the right CS generated by the standard analysis (left) and the new analysis (right) in three individual subjects. The format is similar to Fig. 4. Red curves indicate the approximate location of the dips of the CS.

We applied the boundary mapping methods to the central sulcus (Fig. 6A), in order to inspect whether the boundary mapping methods detected the well-known functional boundary between the primary somatosensory area (S1) and the primary motor area (M1). The delineated boundaries appeared to be located along the fundus of the central sulcus, rather than orthogonally to the sulcus (Fig. 6B). At the same time, the mapping method revealed various probabilistic boundaries other than the putative boundaries between the S1 and M1, indicating the qualitative difference between the classical anatomic areas, such as S1 and M1, and the rsfc-based regions defined in the present study. To evaluate the similarity, the correlation coefficients were calculated between the boundary maps based on whole-brain correlation maps and those based on only a part of the brain in the three cases, and all of these were highly significant (the lowest r = 0.61, t_(140)_ = 9.1, p = 8.7×10^−16^). The correlation coefficients were also calculated between the boundary maps based on the standard analysis using the whole-brain correlation maps and those based on the new analysis in the three cases, and all of these were highly significant (the lowest r = 0.77, t_(130)_ = 13.5, p = 1.6×10^−26^). These results suggest the regional generality of application of the new analysis.

## Discussion

In the present study we developed a new analysis to delineate areal boundaries using signal timecourse within the region of interest, without information about inter-regional interaction in the large-scale networks. It was demonstrated that the pattern of probabilistic boundaries generated by the new method was highly similar to that generated by the standard mapping method. These results suggest that the information for areal boundaries is readily available within the region of interest.

We first tested whether the restricted spatial extent of functional correlation with the seed location, rather than whole-brain correlation, was sufficient for areal boundary mapping using the standard method. It was revealed that the spatial extent of functional correlation to be examined could be restricted to the contralateral part of the region of interest, which contained only 1.5% of the voxels in the whole brain in the present study. Although it is natural that the gray matter of the cerebral cortex, excluding the white matter and the ventricles, was sufficient to draw areal boundaries, these results confirm that the callosal fibers from the region of interest convey rich information on regional interaction to the contralateral region [48], [49]. Examination of functional correlation in the restricted spatial extent should be useful in saving slice coverage during scanning and calculation time in subsequent analyses, compared to the standard method where the whole-brain correlation is to be examined.

The robustness of the areal boundary patterns was also apparent in the results generated by the new method in which the inter-regional correlation was not necessary. The sufficient information about areal boundary that was available in the timecourse within the region of interest suggests that the difference in the timecourses in two adjacent areas depended on the difference in the combination of large-scale brain networks that the two areas belonged to. One caveat regarding the new method is that the resultant probabilistic boundary patterns had a lower contrast, compared to those of the standard analysis, with the boundaries detected by the new method more susceptible to background noise (Figs. 4 and 6). One possible reason would be that the new method cannot utilize the functional contrast that should have been provided by the whole-brain standard method. Moreover, the standard method with correlation calculation only in the contralateral regions had a more efficient signal to noise ratio, with a similar amount of computation time, as compared to the new method (Tables 1 and 2). Although the new method demonstrates that information needed to delineate areal boundaries is readily available inside the region of interest, it does not provide a more effective way of delineating boundaries, except for the cases where, for example, only a limited field of view was scanned for boundary mapping.
